# Aberrant Cerebello-Cerebral Connectivity in Remitted Bipolar Patients 1 and 2: New Insight into Understanding the Cerebellar Role in Mania and Hypomania

**DOI:** 10.1007/s12311-021-01317-9

**Published:** 2021-08-25

**Authors:** Giusy Olivito, Michela Lupo, Andrea Gragnani, Marco Saettoni, Libera Siciliano, Corinna Pancheri, Matteo Panfili, Mara Cercignani, Marco Bozzali, Roberto Delle Chiaie, Maria Leggio

**Affiliations:** 1 Department of Psychology, Sapienza University of Rome, Via Dei Marsi, 78, 00185 Rome, Italy; 2 IRCCS Fondazione Santa Lucia, 00179 Rome, Italy; 3 Servizio di Tutela della Salute Mentale e Riabilitazione dell’Età Evolutiva ASL, Roma 2, 00145 Rome, Italy; 4 Scuola di Psicoterapia Cognitiva SPC, 58100 Grosseto, Italy; 5 Associazione Psicologia Cognitiva (APC)/Scuola di Psicoterapia Cognitiva (SPC), 00185 Rome, Italy; 6 Unità funzionale salute mentale adulti ASL, Toscana nord-ovest, Valle del Serchio, 55100 Pisa, Italy; 7 PhD Program in Behavioral Neuroscience, Sapienza University of Rome, 00185 Rome, Italy; 8 Department of Neuroscience and Mental Health - Policlinico Umberto I Hospital, Sapienza University of Rome, 00161 Rome, Italy; 9 Clinical Imaging Science Center, Brighton and Sussex Medical School, Brighton, East Sussex, BN1 9RR UK; 10 Department of Neuroscience “Rita Levi Montalcini”, University of Turin, 10126 Turin, Italy; 11 Department of Neuroscience, Brighton & Sussex Medical School, University of Sussex, Brighton, East Sussex, BN1 9RR UK

**Keywords:** Cerebellum, Bipolar disorder, Mania, Dentate nucleus, Resting-state fMRI

## Abstract

Bipolar disorder (BD) is a major mental illness characterized by periods of (hypo) mania and depression with inter-episode remission periods. Functional studies in BD have consistently implicated a set of linked cortical and subcortical limbic regions in the pathophysiology of the disorder, also including the cerebellum. However, the cerebellar role in the neurobiology of BD still needs to be clarified. Seventeen euthymic patients with BD type1 (BD1) (mean age/SD, 38.64/13.48; M/F, 9/8) and 13 euthymic patients with BD type 2 (BD2) (mean age/SD, 41.42/14.38; M/F, 6/7) were compared with 37 sex- and age-matched healthy subjects (HS) (mean age/SD, 45.65/14.15; M/F, 15/22). T1 weighted and resting-state functional connectivity (FC) scans were acquired. The left and right dentate nucleus were used as seed regions for the seed based analysis. FC between each seed and the rest of the brain was compared between patients and HS. Correlations between altered cerebello-cerebral connectivity and clinical scores were then investigated. Different patterns of altered dentate-cerebral connectivity were found in BD1 and BD2. Overall, impaired dentate-cerebral connectivity involved regions of the anterior limbic network specifically related to the (hypo)manic states of BD. Cerebello-cerebral connectivity is altered in BD1 and BD2. Interestingly, the fact that these altered FC patterns persist during euthymia, supports the hypothesis that cerebello-cerebral FC changes reflect the neural correlate of subthreshold symptoms, as trait-based pathophysiology and/or compensatory mechanism to maintain a state of euthymia.

**Supplementary Information:**

The online version contains supplementary material available at 10.1007/s12311-021-01317-9.

## Introduction

Bipolar disorder (BD) is a chronic and debilitating illness characterized by episodes of depression and mania (BD type I; BD1) or hypomania (BD type II; BD2), with variable inter-episode remission periods. These conditions can lead to cognitive impairments and changes in sleep, appetite, and psychomotor activity that may persist during euthymic phases [1, 2] with a considerable impact on one’s quality of life. The two subtypes can be distinguished only by elucidating the presence or history of manic or hypomanic episodes, the former characterized by abnormally elevated mood often requiring hospitalization, the latter defined as a less severe type of mania. Although mania and hypomania are the core features that define BD1 and BD2, depressive episodes often exceed manic/hypomanic episodes both in duration and frequency. In spite of the clinical phenomenology that differentiates BD1 and BD2 according to the presence of mania or hypomania, neurobiological studies have not supported this differentiation [3] and neuroimaging data are still not consistent [4]. However, beyond the classification as BD1 and BD2, the main characteristic of the bipolar disorder lies in the peculiar shift into two states with opposing affective valence, i.e., (hypo)mania and depression, that suggests the presence of specific neural mechanisms by which each mood state is triggered [5]. From an anatomical point of view, altered brain network connectivity has been implicated in the pathophysiology of (hypo)manic and depressed states, involving in particular cerebral regions supporting emotion regulation [6, 7] such as frontal, temporal, and limbic regions. Specifically, emotion and cognitive impairments in both symptomatic and euthymic BD could be related to the alteration of the anterior limbic network [6] in which a decreased top-down control of the hypoactive ventral prefrontal areas on limbic brain structures results in hyperactivity of these regions [8].

In the context of mood disorders, the cerebellum has recently gained a great attention in line with its role in emotion and affective processing [9–11]. The hypothesis of cerebellar involvement in mood symptoms of BD is supported by an extensive body of anatomical and clinical research that has widely demonstrated the presence of anatomical connections between cerebellar regions and cerebral associative and subcortical limbic regions implicated in emotional processing and affective behavior [12]. Within the neural networks implicated in BD, the cerebellum has been shown to be closely interconnected with the prefrontal-striatal-circuits and limbic structures, i.e., the amygdala, [6]. Bipolar spectrum disorders have been previously reported in cerebellar patients [13] while aberrant dentate-cortical functional connectivity (FC) has been specifically associated to the onset of a manic state in a patient with an isolated cerebellar lesion [14]. In Lupo and co-authors (2018), the resting-state fMRI (RS-fMRI) analysis evidenced an impaired FC between the cerebellum and the prefrontral-striatal-thalamic network that is typically altered during manic episodes in BD patients. This evidence has been further supported by a recent tractography study showing a greater and widespread cerebro-cerebellar connectivity changes in euthymic BD patients belonging to “mania onset” subphenotype [15]. In spite of this anatomical and clinical evidence, the cerebellar involvement in BD pathophysiology is still poorly understood and needs to be further characterized. Cerebellar structural alterations have been reported in earlier and later stages of BD [3] with no difference between BD1 and BD2 subtypes. Common and different alteration patterns of specific cerebellar lobules have been reported in a recent voxel-based morphometry study (VBM) [16] comparing mixed bipolar and neurodegenerative cerebellar patients, suggesting a cerebellar role in the cognitive and mood dysregulation symptoms that characterize bipolar disorder.

Although the abnormal FC in BD patients has been consistently reported during acute mood episodes [18], a persistent FC vulnerability has been hypothesized also during euthymia when bipolar patients exhibit minimal symptoms by definition [19]. This is probably the result of over-reactive emotional brain networks (i.e., anterior limbic network) that would always leave patients at risk for mood and cognitive disturbances [6–20].

In spite of previous resting-state functional studies that have investigated the cerebello-cerebral connectivity in a mixed sample of remitted BD patients with psychosis [21] and in remitted BD2 patients [22], to our knowledge, this is the first study comparing cerebello-cerebral FC patterns in BD1 and BD2.

Cerebello-cerebral FC changes in BD1 and BD2 during inter-episodic periods will be investigated and characterized by means of RS-fMRI [23]. According to the findings of altered dentate-cerebral connectivity in a cerebellar patient with manic symptoms [14], we ran an a priori hypothesis-driven analysis and used the dentate nucleus (DN), the largest cerebellar output channel, as region of interest (ROI) for the seed-based analysis. The investigation of cerebellar FC patterns in remitted BD patients will aim to elucidate the neural mechanisms involving the cerebellum that are associated to bipolar condition regardless of symptoms presence.

A better definition of the structures involved in BD will result in a deeper comprehension of the pathophysiology of the disorder.

## Material and Methods

### Participants

Seventeen patients with BD type1 (BD1) (mean age/SD, 38.64/13.48; M/F, 9/8) and 13 patients with BD type 2 (BD2) (mean age/SD, 41.42/14.38; M/F, 6/7) were included in the study. All BD patients were recruited from the Department of Psychiatry, Policlinico Umberto I Hospital, and met the Diagnostic and Statistical Manual of Mental Disorders, Fifth Edition (DSM-5) criteria for BD, according to a diagnostic assessment performed with the Italian version of the Structured Clinical Interview for DSM-5 – Clinician Version (SCID-5-CV) [24]. At the time of enrollment, the mean number of years from the first BD diagnosis was 13 (SD 6.4) for BD1 group and 14 (SD 14) for BD2 group. As assessed by the *t*-test and the chi-squared (χ2) analysis, the two groups did not significantly differ in terms of age (*p* > .05) and gender (*p* > .05), respectively. As part of the inclusion criteria, all patients had been euthymic for at least three months. An expert clinical psychiatrist established the euthymic phase by using the Hamilton Depression Rating Scale (HDRS score < 10) [25] and Young Mania Rating Scale (YMRS score < 12) [26]. Other inclusion criteria were (i) first psychiatric examination before the age of 40 years and (ii) suitability for magnetic resonance imaging (MRI). The exclusion criteria for BD patients were (i) other pathological conditions or any cerebral lesion on conventional MRI scans; (ii) other Axis-I psychiatric disorders; (iii) any history of neurological disorders; (iv) mental retardation; and (v) medical condition such as pregnancy, cardiovascular diseases, and diabetes. All patients underwent a neurological evaluation, including the quantification of cerebellar motor deficits using the International Cooperative Ataxia Rating Scale [27] whose global score ranges from 0 (absence of any motor deficit) to 100 (presence of motor deficits at the highest degree).

Clinical characteristics of BD1 and BD2 groups are summarized in Table S1 in Supplementary Material.

All BD participants were under medical treatment. Details of pharmacotherapy for each patient are reported in Table S2 in Supplementary Material.

Thirty-seven sex- and age-matched healthy subjects (HS) with no history of neurological or psychiatric illness (mean age/SD, 45.65/14.15; M/F, 15/22) were recruited as control group. Both BD and control groups did not differ in terms of age and gender distribution as assessed by the *t*-test and the chi-squared (χ^2^) analysis (*p* >.05). This research study was approved by the Ethics Committee of Santa Lucia Foundation according to the principles expressed in the Declaration of Helsinki. Written informed consent was obtained from each subject.

### MRI Acquisition Protocol

All subjects underwent an MRI examination at 3T (Magnetom Allegra, Siemens, Erlangen, Germany) that included the following acquisitions: (1) dual-echo turbo spin echo [TSE] (TR = 6190 ms, TE = 12/109 ms); (2) fast-FLAIR (TR = 8170 ms, 204TE = 96 ms, TI = 2100 ms); (3) T1- weighted 3D high-resolution scan (3D modified driven equilibrium Fourier transform (MDEFT) (TR=1338 ms, TE = 2. 4 ms, mat r i x = 256 × 224 × 176, in- plane FOV = 250 × 250 mm2, slice thickness = 1 mm); (4) T2* weighted echo planar imaging (EPI) sensitized to blood oxygenation-level dependent imaging (BOLD) contrast (TR 2080 ms, TE 30 ms, 32 axial slices parallel to AC-PC line, matrix 64 × 64, pixel size 3 × 3 mm^2^, slice thickness 2.5 mm, flip angle 70°) for resting-state fMRI. BOLD echo planar images were collected during rest for a 7-min and 20-s period, resulting in a total of 220 volumes. During this acquisition, subjects were instructed to keep their eyes closed, not to think of anything in particular, and not to fall asleep. The TSE scans of patients, acquired as part of this research study, were reviewed by an expert neuroradiologist in order to characterize the brain anatomy and ensure the absence of any macroscopic structural abnormalities.

For the HS group, conventional MRI was inspected in order to exclude any pathological conditions according to the inclusion criteria.

### Resting-State fMRI Data Preprocessing

Data were pre-processed using Statistical Parametric Mapping version 8 [Wellcome Department of Imaging Neuroscience; SPM8 (http://www.fil.ion.ucl.ac.uk/spm/)], and in-house software implemented in Matlab (The Mathworks Inc., Natick, Massachussetts, USA). For each subject, the first four volumes of the fMRI series were discarded to allow for T1 equilibration effects. The pre-processing steps included correction for head motion, compensation for slice-dependent time shifts, normalization to the EPI template in MNI coordinates provided with SPM8, and smoothing with a 3D Gaussian Kernel with 8 mm^3^ full width at half maximum. For each data set, motion correction was checked to ensure that the maximum absolute shift did not exceed 2 mm and the maximum absolute rotation did not exceed 1.5°. The global temporal drift was removed using a third-order polynomial fit, and the signal was regressed against the realignment parameters, and the signal averaged over whole brain voxels, to remove other potential sources of bias. Then, all images were filtered by a phase-insensitive band-pass filter (pass band 0.01–0.08 Hz) to reduce the effect of low-frequency drift and high-frequency physiological noise. Every participant’s MDEFT was segmented in SPM in order to estimate the total grey matter (GM) volume to be set as nuisance variable.

### Definition of Regions of Interest and Seed-Based Analyses

**Fig. 1:**
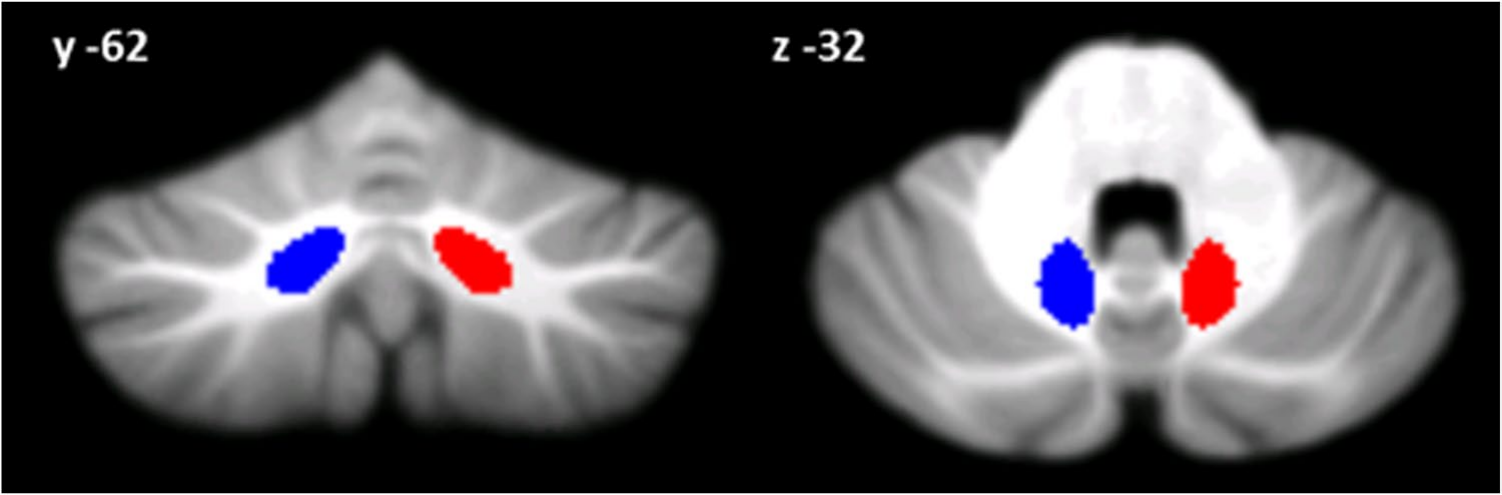
Seed region in the cerebellar dentate nucleus. Coronal (y) and axial (z) view of the generated right (red) and left (blue) dentate nucleus superimposed to the spatially unbiased atlas template of the cerebellum and brainstem (SUIT) (28)

According to the findings of Lupo and colleagues (2018), the DN was chosen as ROI for the RS-fMRI seed-based analysis. The left and right DN masks were separately extracted according to the spatially unbiased atlas template of the cerebellum and brainstem (SUIT) [28] (Fig. 1), resliced into EPI standard space and used as two distinct regions of interest (ROI) for the seed-based analysis. The GM volume of both left and right DN was also calculated for each participant using the command line *“fslstats”* from the FMRIB software library (FSL, www.fmrib.ox.ac.uk/fsl/) applied to the modulated GM maps using the dentate nucleus mask. A one-way ANOVA was performed in Statistical Package for the Social Sciences (SPSS version 25) to test difference among the three groups.

In order to estimate the correlation between each voxel in the brain and the seed regions, we used a first-level SPM model. The mean time course within each seed ROI was extracted for every participant and used as a regressor in a first-level SPM analysis. The resulting beta images are thus equivalent to the Fisher z-transformed maps of the correlation coefficient. These images were taken to the second level, for a group analysis. Specifically, two-sample *t*-test models were used to explore differences in connectivity between each group of patients and controls in each ROI. Additionally, a two-sample *t*-test was performed to investigate differences in cerebello-cerebral FC between BD1 and BD2 groups. No subject showed significant motion. Between-group statistical significance was set at *p* < 0.05 FWE-corrected at cluster level (clusters formed with uncorrected voxels *p* < 0.001 at voxel level). GM volume was set as covariate of no interest.

### Behavioral Correlations with Functional Connectivity

Based on RS-fMRI results, the mean cerebello-cerebral FC values from clusters that were significantly altered in patients were extracted and correlated with mania and depression scores (see Participants sections) as assessed by the HDRS [25] and YMRS [26], respectively. Correlations between behavioral scores and FC value in BD patients were performed by Spearman’s Test by means of SPSS statistics package. To avoid 1 Type error, the Bonferroni correction was applied to correct for multiple testing.

## Results

### Clinical Assessment

BD1 and BD2 did not exhibit (hypo)manic or depressive symptoms according to the HDRS (BD1, mean/SD = 1,00/1,41; BD2, mean/SD = 2,62/3,23) and YMRS (BD1, mean/SD = 1,29/3,06; BD2, mean/SD = 1,77/ 2,74. As assessed by the ICARS scale, both BD1 (mean/SD = 0,88/1,31) and BD2 (mean/SD = 1,17/2,76) patients did not show cerebellar symptoms.

### Seed-Based Analysis

As assessed by the one-way ANOVA, GM volume of both left and right DN did not significantly differ among the three groups (left DN F = 0,879, *p* = 0.420; right DN F = 0,634; *p* = 0.534).

**Fig. 2:**
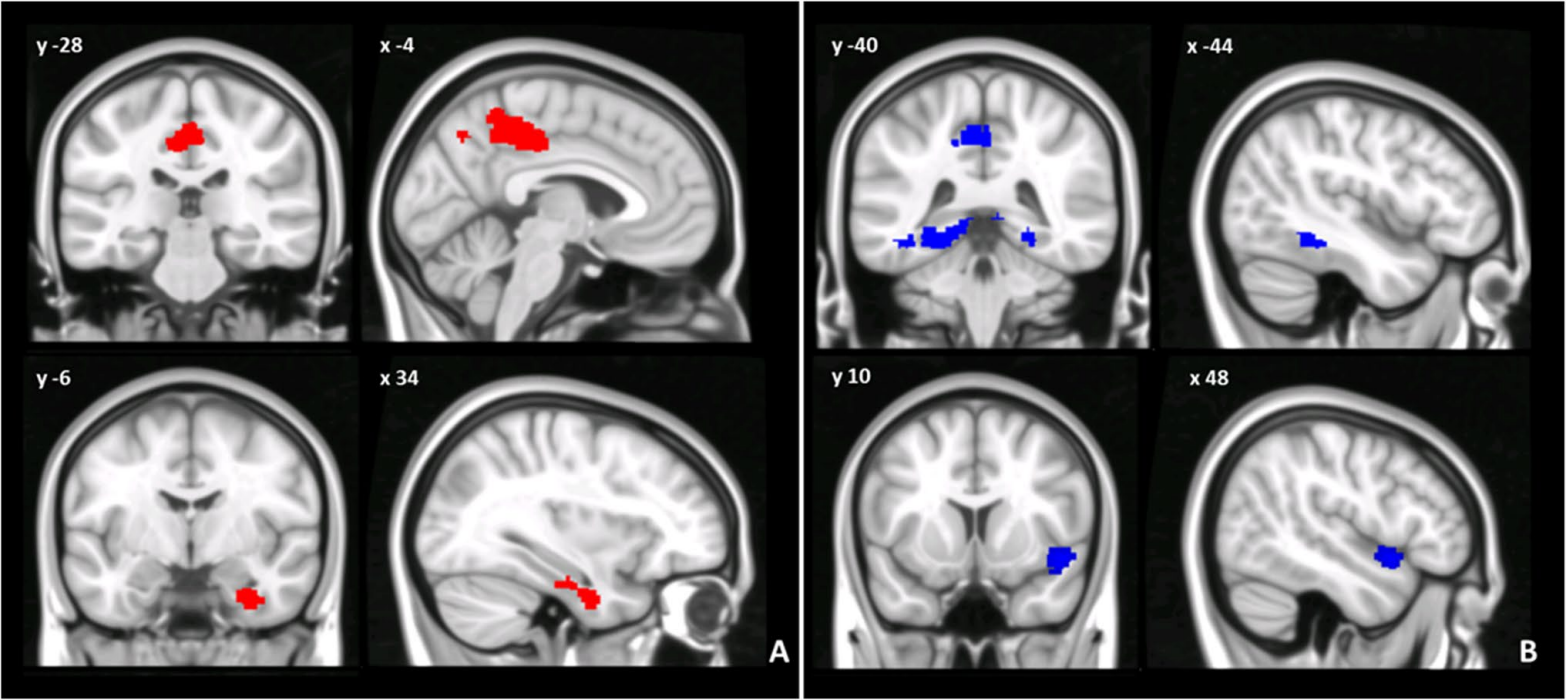
(**A**, **B**) Patterns of dentate functional connectivity with cerebral cortex in BD1. **A** Patterns of increased (top panel) and decreased (bottom panel) FC between right dentate and cerebral cortex in BD1 compared to HS (in red) are shown in coronal (y), and sagittal (x) slices . **B** Patterns of increased (top panel) and decreased (bottom panel) FC between left dentate and cerebral cortex in BD1 compared to HS (in blue) are shown in coronal (y) and sagittal (x) slices. Coordinates (X, Y) are in the Montreal Neurological Institute space. Clusters of altered FC in the cerebral cortex were considered significant after correction for multiple comparisons (FWE corrected *p* < 0.05) Images are shown in neurological convention. See Table 4 for detailed statistics

When compared to controls, BD1 patients showed an altered pattern of dentate-cerebral FC involving both left and right dentate nucleus. Specifically, the left dentate nucleus showed increased FC with left temporal fusiform cortex, right hippocampus, and left posterior cingulate gyrus and decreased FC with the right temporal pole. Additionally, the right dentate nucleus showed increased FC with left posterior cingulate gyrus and decreased FC with right temporal fusiform cortex (Fig. 2A, B). Detailed statistics with peak-voxels showing statistical significance in a cluster are reported in Table 1(A, B).

**Table 1:** Statistics of right (A) and left (B) DN functional connectivity results in BD1

		Cluster size (NoV)	Coordinates			Cluster peak *Z*-score	Brain region
			x	y	z		
**A) R-DN**	BD1 > HS	1485	− 4	− 28	42	5.11	L-posterior cingulate gyrus
			− 20	− 42	44	4.14	
			− 16	− 52	52	4.11	
	BD1 < HS	266	34	− 6	− 34	5.39	R-temporal fusiform cortex
			28	− 14	− 28	4.35	
			34	− 20	− 26	4.06	
**B) L-DN**	BD1 > HS	1758	− 46	− 44	− 10	4.63	L-temporal fusiform cortex
			− 16	− 30	10	4.51	
			− 12	− 12	18	4.48	
		337	34	− 32	− 4	4.43	R-hippocampus
			24	− 44	− 8	3.91	
			14	− 48	− 6	3.86	
		277	− 4	− 30	44	4.18	L-posterior cingulate gyrus
			− 4	− 40	46	3.86	
			− 6	− 54	50	3.41	
	BD1 < HS	199	48	10	− 12	3-87	R-temporal pole

Significant increased (BD1 > HS) and decreased (BD1 < HS) dentate FC in BD1 compared to HS are reported. MNI coordinates (*x, y, z*) in the Montreal Neurological Institute space and peak Z-score of the peak voxels showing greatest statistical differences in a cluster are reported. Only regions that survived after correction for multiple comparisons (FWE corrected *p* < 0.05) have been considered. *NoV*, number of voxels; *L*, left; *R*, right

**Fig. 3:**
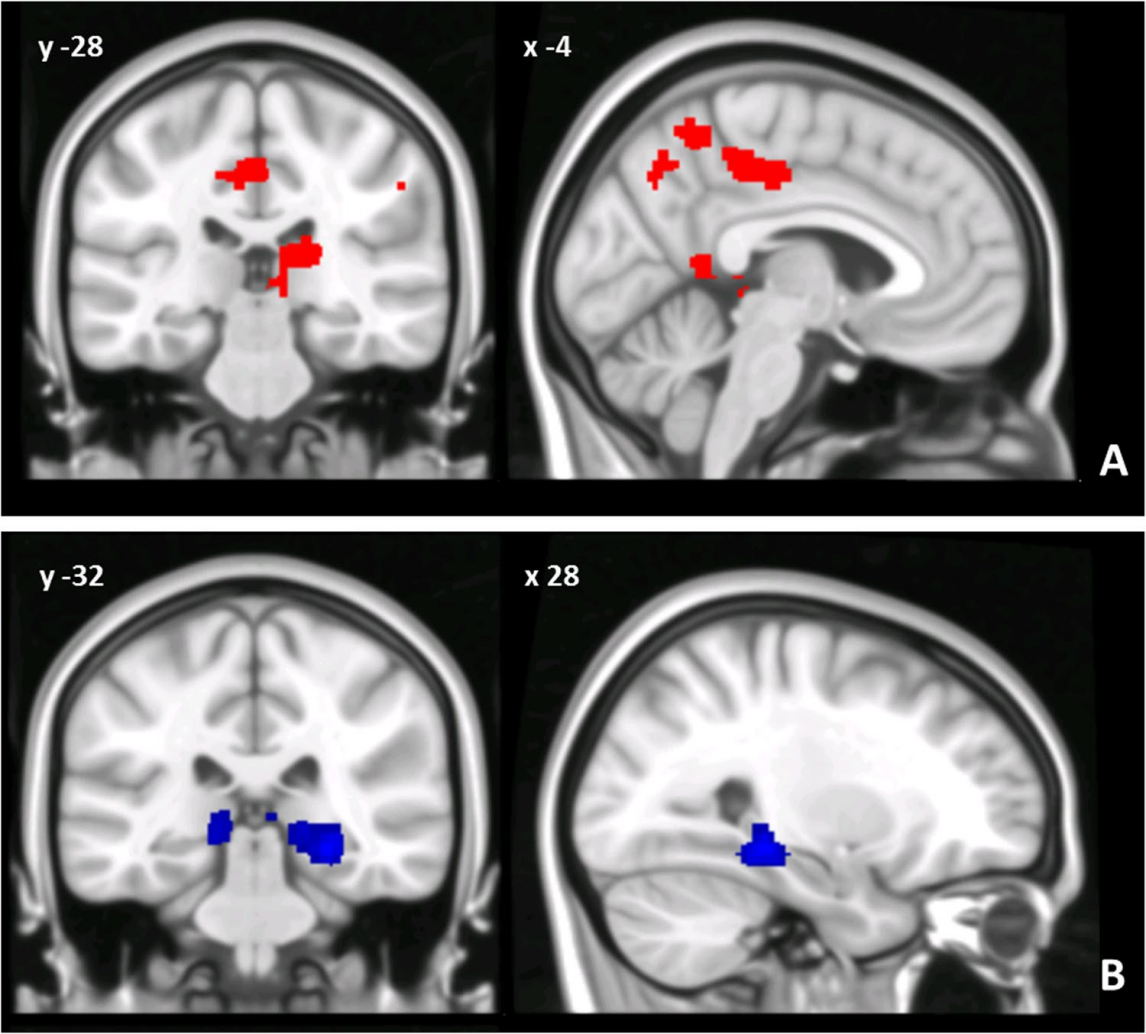
(**A**, **B**) Patterns of dentate functional connectivity with cerebral cortex in BD2. **A** Patterns of increased FC between right dentate and cerebral cortex in BD2 compared to HS (in red) are shown in coronal (y) and sagittal (x) slices. **B** Patterns of increased FC between left dentate and cerebral cortex in BD1 compared to HS (in blue) are shown in coronal (y) and sagittal (x) slices. Coordinates (X, Y) are in the Montreal Neurological Institute space. No patterns of decreased FC were evidenced in BD2 compared to HS. Clusters of altered FC in the cerebral cortex were considered significant after correction for multiple comparisons (FWE corrected *p* < 0.05). Images are shown in neurological convention. See Table 5 for detailed statistics

When compared to controls, BD2 patients also showed an altered pattern of cerebello-cerebral FC involving both left and right dentate nucleus. Specifically, the left dentate nucleus showed increased FC with the right parahippocampal gyrus and right lateral occipital cortex, while no decreased FC was evidenced. Similarly, the right dentate nucleus showed increased FC with the left posterior cingulate gyrus, the right pulvinar, and the right angular gyrus while no decreased FC was evidenced (Fig. 3A, B). Finally, no significant differences in dentate-cerebral FC were found between BD1 and BD2 groups.

Detailed statistics with peak-voxels showing statistical significance in a cluster are reported in Table 2(A, B).

**Table 2:** Statistics of right (A) and left (B) DN functional connectivity results in BD2

		Cluster size (NoV)	Coordinates			Cluster peak *Z*-score	Brain region
			x	y	z		
**A) R-DN**	**BD2 > HS**	743	− 4	− 28	42	4.80	L-posterior cingulate gyrus
			0	38	46	4.18	
			− 4	− 52	58	4.18	
		443	20	− 26	12	4.56	R-thalamus
			12	− 22	12	4.22	
			4	− 52	4	3.85	
		244	44	− 48	52	3.78	R-angular gyrus
			48	− 38	50	3.54	
			54	− 30	40	3.40	
**B) L-DN**	**BD2 > HS**	2077	28	− 32	− 10	4.62	R-parahippocampal gyrus
			− 12	− 62	52	4.53	
			− 20	− 40	− 14	4.21	
		220	48	− 64	34	4.11	R-lateral occipital cortex
			46	− 64	46	3.68	
			38	− 64	50	3.86	

Significant increased (BD2 > HS) dentate FC in BD2 compared to HS is reported. MNI coordinates (*x, y, z*) in the Montreal Neurological Institute space and peak Z-score of the peak voxels showing greatest statistical differences in a cluster are reported. Only regions that survived after correction for multiple comparisons (FWE corrected *p* < 0.05) have been considered. *NoV*, number of voxels; *L*, left; *R*, right

### Behavioral Correlations

Spearman’s correlations coefficients revealed a pattern of correlation between mania scores and different cerebral regions of altered FC in both groups. Specifically, BD1 patients showed significant negative correlations between mania scores and decreased FC between right dentate nucleus and right temporal fusiform cortex (R = − 0.533 *p* = < 0.02). Conversely, BD2 patients showed significant negative correlations between mania scores and increased FC between right dentate nucleus and left cingulate gyrus (R = − 0.634 *p* = < 0.02) and right angular gyrus (R = − 0.615 *p* = < 0.02). However, after applying the Bonferroni correction to control for the family-wise error rate, the association between mania scores and altered FC was no longer statistically significant in both groups.

No correlations between depression scores and FC patterns were detected.

Detailed statistics for both YMRS and HDRS correlations are reported in Table S3 in Supplementary materials.

## Discussion

This is a preliminary study aiming to investigate the cerebellar role in the pathophysiology of BD1 and BD2. To this aim, we assessed cerebello-cerebral FC patterns that are associated with BD1 and BD2 during remission stage. As previously stated, the investigation of resting–state FC during euthymia allows to highlight cerebral regions that present persistent functional changes regardless of symptoms presence thus reflecting the neural correlate of subthreshold symptoms, as trait alterations independent of mood state or a functional state adopted to maintain the clinical remission.

When compared to HS, we found patterns of altered cerebello-cerebral FC in the two BD subgroups. Generally speaking, BD1 showed a pattern of both hyper- and hypo-connectivity between the cerebellum and regions of the cerebral cortex, while BD2 only showed cerebello-cerebral hyper-connectivity. In BD1 subgroup, decreased dentate connectivity was found with the right temporal pole and the right temporal fusiform cortex, while increased dentate connectivity was found with the left temporal fusiform cortex, right hippocampus and left posterior cingulate cortex. In BD2 subgroup, increased dentate connectivity was found with the right parahippocampal gyrus, right lateral occipital cortex, the left posterior cingulate gyrus, the right pulvinar, and the right angular gyrus. Consistent with the present findings, an altered set of regions has been consistently implicated in the mood dysregulation associated to BD condition. The disruption of ventrolateral prefrontal-amygdala emotional pathway has been specifically related to the manic states of BD [6, 29]. In manic BD patients, decreased activation has been reported in ventral prefrontal cortex, cingulate cortex, and striatum, thus leading to hyper-activity of limbic brain areas, such as the amygdala [4, 8, 30]. Closely interconnected to the amygdala, the hippocampal and parahippocampal regions [29, 31] project into the prefrontal-striatal-thalamic circuit that modulates anterior limbic structures [32, 33] and is typically altered during manic episodes in BD patients [32]. It is worth noting that, within this network, the thalamus, and specifically the pulvinar nucleus, is directly connected to important limbic structure including the amygdala and cingulate cortex and relays information related to the emotional content of the environment into the limbic system [34]. Regional differences in subcortical and medial temporal regions, which are all part of the anterior limbic network, have been reported in BD [32, 35]. In particular, dysfunctional connectivity between the fusiform cortex and amygdala has been related to impaired emotional encoding in BD [36]. As part of the Default Mode Network (DMN), the posterior cingulate cortex has been also implicated in BD [37] in line with its crucial role in motivation and drives [38]. Taken together, imaging studies have highlighted that BD does not result from abnormalities within a single neuroanatomic structure, but it is rather the result of an impaired complex system of interconnected neural networks. For a schematic model of interconnected cortical and subcortical regions that have been related to the expression of BD, see Fig. 1 in Strakowski and colleagues (2005).

In this framework, our findings provide novel insight into understanding specific cerebellar contribution to the underlying neuropathological mechanisms in remitted BD1 and BD2 patients.

Indeed, the pattern of cerebello-cerebral alterations in our cohorts suggests an impaired interaction between the cerebellum and specific cerebral regions of the anterior limbic network implicated in (hypo)manic symptoms of BD1 and BD2 [29, 32] that show functional alterations throughout the course of illness.

The cerebellar contribution to mood regulation is now widely accepted. As reported in the cerebellar cognitive-affective syndrome (CCAS) [9], cerebellar alterations have been related to mood disturbances following both pure cerebellar lesion and neurodegenerative cerebellar pathologies [14, 39]. Cerebellar structural alterations have been extensively reported in BD [3, 16, 40]. From an anatomical point of view, this is supported by the close cerebellar connections with the prefrontal-striatal-circuits and limbic structures, i.e., the amygdala and the hippocampus [41]. Furthermore, the participation of the cerebellum to DMN has been widely demonstrated [42, 43]. As regards the cerebellum, traditionally, the emotional processing involves the vermis and fastigial nuclei. While most functional studies have focused on the cerebellar cortex [12], the measurement of activity in the deep cerebellar nuclei, which should relate more to the cerebellar output, could provide useful insights in functional studies. However, small activated regions, as the fastigial nuclei, usually have a low BOLD signal and pose a challenge in fMRI analysis. Furthermore, in a previous functional study, Lupo and colleagues (2018) [14] showed altered cerebello-cerebellar functional connectivity in a patient with a cerebellar lesion involving in particular the dentate nucleus and both cortical (i.e., the dorsolateral prefrontal and orbitofrontal cortex) and subcortical limbic regions (i.e., the cingulate cortex) related to emotional and affective processing.

As the greatest deep cerebellar nucleus, the DN represents the main cerebellar output channel to cerebral cortex. Indeed, the cortical cerebellar outputs converge onto DN, that, in turn, sends neural signals back to the cerebral cortex [44]. According to all these observations, the hypothesis is that the cerebellar structural alterations observed in BD [3, 45] may impact the functional activity of the DN and its modulation of target areas in the cerebral cortex, such as prefrontal, posterior parietal, and limbic regions [46]. Thus, cerebellar dysfunction may contribute to the well-known alterations of these higher-level regions in both BD1 and BD2 and may result in abnormal signal processing of affective, emotional, and behavioral information leading to (hypo)manic symptoms of BD1 and BD2 [14].

Overall, abnormal connectivity between the cerebellum and the described cerebral regions may reflect neural correlate of subthreshold (hypo) manic symptoms that is independent from the mood state and that persists during remission serving as a hallmark of bipolar euthymia. The lack of correlations between altered FC and mania scores provides further support to this hypothesis.

Interestingly, the patterns of cerebello-cerebellar FC changes in BD1 and BD2 may reflect pathological mechanisms characterizing the two groups during euthymia. When compared to controls, hyper- and hypoconnectivity changes were detected in BD1 group, while hyperconnectivity changes were only evidenced in BD2 groups. A possible explanation for this difference can be found in the clinical features that characterize the two subtypes.

Indeed, BD2 patients, by definition, experience less intense manic state (i.e., hypomania) than BD1 (i.e., mania), but their symptoms are more severe and malignant with respect to episode frequency and course [47]. As a result, the different intensity and chronicity of the (hypo)manic symptoms may reflect different FC vulnerability or require different mechanisms in order to maintain a state of euthymia. Further investigations are needed to address this issue and clarify such a differentiation. To conclude, some issues need to be discussed. Firstly, it has to be underlined that we did not find difference in cerebellar FC between BD1 and BD2 subtypes. These findings are in line with a previous whole-brain VBM study showing no structural differences between the two subtypes [3]. In spite of the different FC changes in comparison with healthy subjects, the lack of significant functional differences between the two groups allows to hypothesize that functional differences could be specifically related to acute affective periods and not be detected during the remission stages.

Secondly, it has to be mentioned that left and right DN impaired FC involves both ipsilateral and contralateral regions in the cerebral cortex. However, although the cerebello-cortical connections are known to be mainly contralateral [44], it has been shown that FC may be at least in part independent from effective structural connections [48].

Our investigation has some limitations. The most important limitation is related to the small and unequal sample size of BD1 and BD2 groups. While the present preliminary results need to be confirmed and replicated with greater samples, it has to be underlined that this is the first study investigating and comparing the pattern of cerebello-cerebral FC between well-characterized samples of BD1 and BD2 during euthymia. In spite of the small and unequal samples size, the consistence with the existing literature provides support to our conclusions. Another important limitation is related to the presence of pharmacotherapy since all patients were treated due to the difficulty in finding un-medicated BD patients in remission. Indeed, most people with BD need to manage their condition pharmacologically in order to achieve a clinical stability, so studies involving euthymic participants typically recruit people on medications. In spite of this, previous evidence has showed that pharmacological treatment does not affect the cerebellar structures [49] and FC results [50]. Finally, since no further neuropsychological data were available to match healthy subjects with the BD patients in terms of intelligence, short-term/working/episodic memory, attention/executive function, it has to be taken into account that the resting-state functional connectivity differences reported between the groups could partially reflect group-level differences in these domains.

In terms of possible future developments, the present findings provide a breeding ground for a better pathophysiological comprehension of BD. Future studies with larger patients’ sample may prove whether the cerebellum has the potential to be the target of alternative therapeutic approach to BD 1 and BD2 (hypo)manic symptoms, i.e., the cerebellar neuromodulation.

## Conclusion

In conclusion, the present work provides evidence that the cerebellum contributes to the pathophysiology of BD1 and BD2. Interestingly, the pattern of functional cerebello-cerebral alterations suggests an impaired cerebellar modulation on cerebral regions implicated in (hypo)manic symptoms of BD1 and BD2 that persist under clinical remission stage.

In particular, changes in resting state functional connectivity of the cerebellum and connected cerebral regions in BD1 and BD2 may reflect trait-based pathophysiology or act as compensatory mechanisms responsible for maintaining a state of clinical remission. In terms of possible future developments, the present findings provide a breeding ground for a better pathophysiological comprehension of bipolar disorder as well as for alternative therapeutic approaches specifically targeting the cerebellum.

## Supplementary Information

Below is the link to the electronic supplementary material.

Supplementary file1 (DOCX 22 KB)

Supplementary file2 (DOCX 19 KB)

Supplementary file3 (DOCX 17 KB)
