# Supplementary material for: Aberrant Cerebello-Cerebral Connectivity in Remitted Bipolar Patients 1 and 2: New Insight into Understanding the Cerebellar Role in Mania and Hypomania
Source: Cerebellum. 2021 Aug 25;21(4):647–56. doi: 10.1007/s12311-021-01317-9 (PMC9325834; doi:10.1007/s12311-021-01317-9)
Supplement: Supplementary file 1 — Supplementary file1 (DOCX 22 KB) [file 12311_2021_1317_MOESM1_ESM.docx]

**Table S1. Clinical details of BD1 and BD2 groups.** Number of total episodes and present symptoms are reported for each subject of BD1 and BD2 groups. *(precipitating factors that have determined a symptomatic crisis, such as: bereavement in the family, layoff, divorce, end of a relationship or illness)

|  | Number of Total Episode | | | | | Present symptoms (Yes or No) | | | | |
| --- | --- | --- | --- | --- | --- | --- | --- | --- | --- | --- |
| ID | Manic | Hypomanic | Depressive | Mixed | Hospitalization | Delusions | Hallucinations | Sleep disorders | Suicidal thoughts/ and Suicides attempts | Stressful Events* |
| BD1-1 | 4 | 0 | 0 | 1 | 5 | Y | N | N | N | Y |
| BD1-2 | 1 | 0 | 2 | 0 | 1 | Y | N | N | N | N |
| BD1-3 | 0 | 0 | 1 | 13 | 13 | Y | Y | N | Y/Y | N |
| BD1-4 | 3 | 0 | 1 | 0 | 2 | N | N | N | N | N |
| BD1-5 | 0 | 0 | 1 | 3 | 3 | Y | Y | N | Y/Y | Y |
| BD1-6 | 0 | 2 | 1 | 1 | 1 | N | N | Y | Y/Y | Y |
| BD1-7 | 1 | 1 | 2 | 0 | 2 | N | N | N | Y/Y | Y |
| BD1-8 | 2 | 0 | 2 | 2 | 6 | Y | N | N | Y/Y | Y |
| BD1-9 | 2 | 0 | 0 | 0 | 2 | N | N | N | N | Y |
| BD1-10 | 4 | 1 | 3 | 0 | 2 | Y | N | N | N | N |
| BD1-11 | 3 | 0 | 2 | 0 | 4 | Y | N | N | N | Y |
| BD1-12 | 2 | 0 | 3 | 0 | 0 | Y | Y | Y | N | Y |
| BD1-13 | 3 | 1 | 1 | 1 | 2 | Y | N | N | N | N |
| BD1-14 | 1 | 2 | 1 | 1 | 1 | Y | Y | N | N | Y |
| BD1-15 | 1 | 0 | 1 | 1 | 1 | N | N | N | Y/Y | N |
| BD1-16 | 3 | 0 | 0 | 1 | 3 | Y | Y | N | N | N |
| BD1-17 | 2 | 2 | 1 | 1 | 2 | Y | Y | N | Y/Y | Y |
| BD2-1 | 0 | 0 | 0 | 2 | 0 | N | N | N | N/N | Y |
| BD2-2 | 0 | 0 | 2 | 0 | 0 | N | N | N | N/N | Y |
| BD2-3 | 1 | 1 | 3 | 1 | 1 | N | N | Y | N/N | N |
| BD2-4 | 0 | 0 | 1 | 1 | 0 | N | N | N | N/N | Y |
| BD2-5 | 0 | 2 | 1 | 0 | 0 | N | N | Y | N/N | Y |
| BD2-6 | 0 | 3 | 2 | 0 | 0 | N | N | N | N/N | N |
| BD2-7 | 0 | 0 | 1 | 1 | 2 | N | N | N | N/N | N |
| BD2-8 | 0 | 0 | 0 | 1 | 1 | N | N | N | Y/N | Y |
| BD2-9 | 0 | 1 | 2 | 0 | 1 | N | N | N | N/N | Y |
| BD2-10 | 0 | 2 | 3 | 0 | 0 | N | N | N | Y/N | N |
| BD2-11 | 0 | 1 | 1 | 0 | 0 | N | N | N | N/N | Y |
| BD2-12 | 0 | 0 | 0 | 1 | 0 | N | N | N | N/N | N |
| BD2-13 | 3 | 0 | 2 | 0 | 3 | Y | N | N | N/N | Y |
