# Supplementary material for: Aberrant Cerebello-Cerebral Connectivity in Remitted Bipolar Patients 1 and 2: New Insight into Understanding the Cerebellar Role in Mania and Hypomania
Source: Cerebellum. 2021 Aug 25;21(4):647–56. doi: 10.1007/s12311-021-01317-9 (PMC9325834; doi:10.1007/s12311-021-01317-9)
Supplement: Supplementary file 2 — Supplementary file2 (DOCX 19 KB) [file 12311_2021_1317_MOESM2_ESM.docx]

**Table S2. Current pharmacotherapy of BD1 and BD2 groups.** Pharmacotherapy details are reported for each subject of both BD1 and BD2 groups.

| ID | Antipsychotics | Lithium | Antiepileptics | Antidepressants | Anxiolytic | Polypharmacy |
| --- | --- | --- | --- | --- | --- | --- |
| BD1-1 | X |  |  |  |  |  |
| BD1-2 | X |  | X |  |  | X |
| BD1-3 |  | X | X |  | X | X |
| BD1-4 | X |  | X | X |  | X |
| BD1-5 | X | X |  |  | X | X |
| BD1-6 |  |  | X |  |  |  |
| BD1-7 |  | X | X |  |  | X |
| BD1-8 | X | X | X | X |  | X |
| BD1-9 | X | X |  |  |  | X |
| BD1-10 | X | X | X |  |  | X |
| BD1-11 | X |  | X |  |  | X |
| BD1-12 | X |  | X |  |  | X |
| BD1-13 | X | X | X |  |  | X |
| BD1-14 | X | X | X |  |  | X |
| BD1-15 |  | X | X |  |  | X |
| BD1-16 | X |  | X |  |  | X |
| BD1-17 |  | X |  |  |  |  |
| BD2-1 | X |  | X |  | X | X |
| BD2-2 | X | X | X |  | X | X |
| BD2-3 |  | X | X |  |  | X |
| BD2-4 |  |  | X |  |  |  |
| BD2-5 |  |  | X |  |  |  |
| BD2-6 | X | X | X |  |  | X |
| BD2-7 | X | X | X |  |  | X |
| BD2-8 |  | X |  |  |  |  |
| BD2-9 | X | X | X |  |  | X |
| BD2-10 |  |  |  | X |  |  |
| BD2-11 | X |  | X |  | X | X |
| BD2-12 |  |  | X |  |  |  |
| BD2-13 | X | X |  |  |  | X |
