# Supplementary material for: Aberrant Cerebello-Cerebral Connectivity in Remitted Bipolar Patients 1 and 2: New Insight into Understanding the Cerebellar Role in Mania and Hypomania
Source: Cerebellum. 2021 Aug 25;21(4):647–56. doi: 10.1007/s12311-021-01317-9 (PMC9325834; doi:10.1007/s12311-021-01317-9)
Supplement: Supplementary file 3 — Supplementary file3 (DOCX 17 KB) [file 12311_2021_1317_MOESM3_ESM.docx]

| **Group** | **Cerebellar seed** | **Cerebral Regions** | **HDRS** | **YMRS** |
| --- | --- | --- | --- | --- |
| **BD1** | ***R-DN*** | L-Cingulate | R = 0,21; p = 0.42 | R = 0.35; p = 0.16 |
|  |  | R-Temporal Fusiform | R = -0.48; p = 0.06 | R = -0.53; p = 0.02* |
|  | ***L-DN*** | L-Temporal Fusiform | R = 0.32; p = 0.22 | R = 0.19; p = 0.44 |
|  |  | R-Hippocampus | R = -0.19; p = 0.46 | R = 0.07; p = 0.76 |
|  |  | L-Cingulate | R = -0.00; p = 0.98 | R = 0.45; p = 0.06 |
|  |  | R-Temporal Pole | R = -0.30; p = 0.24 | R = -0.47; p = 0.05 |
| **BD2** | ***R-DN*** | L-Cingulate | R = 0.10; p = 0.72 | R = -0.63; p = 0.02* |
|  |  | R-Thalamus | R = 0.04; p = 0.88 | R = 0.13; p = 0.65 |
|  |  | R-Angular Gyrus | R = -0.24; p = 0.41 | R = -0.61; p = 0.02* |
|  | ***L-DN*** | R-Parahippocampal Gyrus | R = -0.18; p = 0.54 | R =- 0.04; p = 0.89 |
|  |  | R-Lateral Occipital cortex | R = 0.24; p = 0.41 | R = 0.22; p = 0.45 |

**Table S3. Correlations between impaired dentate-cerebral FC and HDRS and YMRS scores in BD1 and BD2 patients.**

*correlations significant at p=<0.05; BD: Bipolar Disorder; DN: Dentate Nucleus; L=left; R:right.

HDRS: Hamilton Depression Rating Scale (Hamilton, 1967); YMRS: Young Mania Rating Scale (Young et al., 1978).
